# Roles for RNA export factor, *Nxt1*, in ensuring muscle integrity and normal RNA expression in *Drosophila*

**DOI:** 10.1093/g3journal/jkaa046

**Published:** 2020-12-24

**Authors:** Kevin van der Graaf, Katia Jindrich, Robert Mitchell, Helen White-Cooper

**Affiliations:** School of Biosciences, Cardiff University, Cardiff CF10 3AT, UK

**Keywords:** *Drosophila*, RNA export pathway, muscle maintenance, circRNA

## Abstract

The mRNA export pathway is responsible for the transport of mRNAs from the nucleus to the cytoplasm, and thus is essential for protein production and normal cellular functions. A partial loss of function allele of the mRNA export factor *Nxt1* in *Drosophila* shows reduced viability and sterility. A previous study has shown that the male fertility defect is due to a defect in transcription and RNA stability, indicating the potential for this pathway to be implicated in processes beyond the known mRNA transport function. Here we investigate the reduced viability of *Nxt1* partial loss of function mutants, and describe a defect in growth and maintenance of the larval muscles, leading to muscle degeneration. RNA-seq revealed reduced expression of a set of mRNAs, particularly from genes with long introns in *Nxt1* mutant carcass. We detected differential expression of circRNA, and significantly fewer distinct circRNAs expressed in the mutants. Despite the widespread defects in gene expression, muscle degeneration was rescued by increased expression of the costamere component *tn (abba)* in muscles. This is the first report of a role for the RNA export pathway gene *Nxt1* in the maintenance of muscle integrity. Our data also links the mRNA export pathway to a specific role in the expression of mRNA and circRNA from common precursor genes, *in vivo.*

## Introduction

Regulation of mRNA expression depends on transcriptional regulatory processes, such as transcription factor and repressor binding to DNA. Post-transcriptional regulation of RNA adds additional layers of potential control, both in the nucleus and after mRNA export to the cytoplasm. Within the nucleus, critical processing and control points include (alternative) splicing of exons and association of the RNA with mRNA nuclear export factors. The recognition of mRNAs as nuclear export cargoes by the nuclear pores depends on the binding of the Nxf1/Nxt1 protein heterodimer, which directly interacts with nuclear pore components (reviewed in Xie and Ren 2019). Recruitment of Nxf1/Nxt1 depends on appropriate processing of the RNAs within the nucleus (reviewed in Williams *et al.* 2018). The highly conserved transcription-export complex (TREX), which itself comprises a THO subcomplex associated with a helicase (UAP56) and an adapter (ALYREF, Ref1 in *Drosophila*), associates with RNAs as they are being transcribed (Sträßer *et al.* 2002). For intron-containing transcripts, recruitment of the spliceosome, and subsequent completion of splicing of each intron, results in deposition of the exon junction complex (EJC) at the splice site (Le Hir *et al.* 2000). This facilitates TREX recruitment (Le Hir *et al.* 2001), although TREX recruitment also occurs independently of splicing, mediated both by cap-dependent interactions, and interactions between ALYREF and the body of transcripts (Cheng *et al.* 2006; Chi *et al.* 2013; Viphakone *et al.* 2019). ALYREF acts to stimulate the RNA-binding activity of Nxf1, and thus ensures that the Nxf1/Nxt1 heterodimer is recruited to the mRNA, permitting its subsequent export. Recruitment of export factors occurs co-transcriptionally, and is typically completed before the final act of mRNA production, namely cleavage and polyadenlyation.

We have previously described a hypomorphic allele of *Nxt1* in *Drosophila* (Caporilli *et al.* 2013). These flies express a mutant Nxt1 protein that has reduced stability, but sufficient function remains to allow normal mRNA nuclear export. This allele therefore allows us to investigate any additional roles for Nxt1, and the RNA export pathway, *in vivo*. Investigation of the male sterility caused by this mutant revealed that *Nxt1* is critical for male fertility: it is required for normal levels of transcription of a specific subset of testis-expressed genes, and crucially for the stability of nascent transcripts from these genes (Caporilli *et al.* 2013). This is consistent with Nxt1 being required *in vivo* for regulation of additional RNA-processing functions in addition to its key role in mRNA nuclear export.

The formation, growth, and maintenance of the musculature are critical for normal animal function, and defects in these processes can lead to impaired mobility, shorter lifespan, or early lethality. The somatic muscular tissue of *Drosophila melanogaster* is generated via a highly regulated developmental programme in embryogenesis, such that the first instar larva contains a stereotyped pattern of muscles (Bate 1990). Each muscle is a single multinucleated cell, derived from the fusion of muscle founder cells with fusion-competent myoblasts (reviewed in Kim *et al.* 2015). Very severe defects in embryonic myogenesis result in a failure of the embryo to hatch. Less severe defects in the development of the muscle pattern or in muscle function can result in animals which are viable, but with impaired mobility. During larval stages, no new cells are added but the muscles grow extensively by the addition of new myofibrils, particularly during the final larval instar (reviewed in Piccirillo *et al.* 2014). Normal muscle function is essential during pupariation for contraction, spiracle eversion, and air bubble migration (Shatoury 1959), and defects in larval muscles can lead to gross morphological defects and lethality at this stage.

While most pre-mRNAs are spliced to generate linear mRNA molecules, a small proportion generating circular RNAs (circRNAs) by splicosome-dependent back splicing of segments of primary transcripts (reviewed in Huang *et al.* 2017; Li *et al.* 2018; Patop *et al.* 2019). The 3' end of an exon splices to the 5' end of the same exon, or to a further upstream splice acceptor site in some cases. The production of circRNA depends on alternative transcript processing; typically, the exon that is circularized is not subject to conventional alternative splicing, but is flanked by relatively long introns (Jeck *et al.* 2013). Modulation of transcription rate and splicing rate both affect the relative efficiency of forward and back splicing events, and thus can alter the expression level or ratio of the alternative products (Ashwal-Fluss *et al.* 2014; Zhang *et al.* 2016). Once formed, circRNAs are relatively stable, and, while typically of relatively low abundance, they can accumulate to levels equal to or even exceeding that of the mRNA(s) derived from the same gene. At least 2500 distinct circRNAs are expressed in *Drosophila melanogaster* (Westholm *et al.* 2014). CircRNAs are particularly abundant in neural tissue, but all analyzed tissues express some circRNAs (Westholm *et al.* 2014). CircRNAs have been shown to have a variety of roles *in vivo*, including acting as miRNA sponges, protein sponges, protein scaffolders, and being translated (reviewed in Huang *et al.* 2017; Li *et al.* 2018).

Here, we use the partial loss of function allele to investigate functions of the RNA export pathway in addition to export itself and determine two distinct phenotypic effects. At a morphological level, we describe a specific role for Nxt1 in the maintenance of larval muscles. We determined that *Nxt1* partial loss of function animals showed muscle degeneration during the extensive growth associated with the final larval instar stage. Knock down of other RNA export factors caused a similar muscle degeneration phenotype. Genome-wide molecular analysis of RNAs revealed that normal expression of many genes, particularly those with long introns that are sources of circRNAs, requires *Nxt1*. We discovered that both the mRNA and circRNA products of many of these *Nxt1*-responsive genes were reduced in mutants, although the nascent transcripts of these genes were typically produced at normal levels.

Despite the large number of altered transcripts, we were able to rescue the muscle degeneration of *Nxt1* mutants, but not the pupal lethality, by expression of the *Drosophila* homologue of *Trim32*, *tn (abba)*, in muscles. This suggests that the muscle degeneration is probably caused by the defects in RNA processing, particularly the reduction in abba mRNA. The finding that *abba* re-expression is not sufficient to rescue the reduced viability of the *Nxt1* hypomorphs is consistent with the defects in expression of many other genes, whose normal expression is presumably required for full viability. Together, our data indicate an unexpected role for *Nxt1* in post-transcriptional regulation of many mRNAs and circRNA splice products, and show that this RNA export protein is essential for muscle maintenance.

## Materials and methods

### *Drosophila* culture, strains, genetics, mobility, and viability assays

Flies were maintained in cornmeal, yeast, dextrose, agar medium, at 25**°**C unless otherwise stated. *w^1118^* was used as a control. *Nxt1^z2-0488^, Nxt1 DG05102*, and UAS-eGFP-Nxt1 are as described in Caporilli *et al.* (2013). VDRC RNAi lines were KK107745 (*Nxt1*), P{GD17336}v52631 (*Nxt1*) KK102231 (*thoc5*), KK100882 (*Nxf1*), and KK109076 (Ref1) (Dietzl *et al.* 2007). w; UAS-*abba^fl^* was from (Domsch *et al.* 2013); Mef2-Gal4 from (Ranganayakulu *et al.* 1996). Arm-Gal4 and UAS-dicer were from BDSC. For larval starvation, larvae were removed from food and maintained on damp filter paper in a petri dish. To assay mobility, larvae were filmed for 200 s at 1 fps while crawling on an agar dish. To assay survival from larval to adult stages, larvae were placed on fresh food and examined daily.

### RNA extraction, cDNA synthesis, and quantitative PCR

Total RNA was extracted using Trizol (ThermoFisher Scientific) followed by the RNeasy Mini Kit (Qiagen). cDNA was generated using 100 ng total RNA and oligo dT or random hexamer primers with the Superscript III kit (Invitrogen). qRT-PCR was performed using PowerSybr reagent (ABI) or FastStart essential DNA green master mix (Roche); *Rp49* was used as a control gene for normalization. Sequences of all primers are given in Supplementary Table S1.

### Dissection, staining, imaging, and scoring of muscle defects

Pupae were prepared for scanning electron microscopy by sputter coating with Au90Pd10 and imaged with in a FEI-XL30 Field Emission Gun Environmental Scanning Electron Microscope. Larvae were dissected in low Ca^2+^ saline, HL-3 (Pesavento *et al.* 1994) in a magnetic chamber (Budnik *et al.* 2006), and fixed in 4% paraformaldehyde in PBS for 1 h. F-actin was labeled with Alexa Fluor^TM^ 488 Phalloidin (ThermoFisher). All 30 muscles in 8 hemisegments (A2–A5) were scored each larva (240 muscles per animal) for signs of reduced integrity (torn, thin, loss of sarcomeric structure, or missing).

### RNA sequencing and analysis

For whole larva sequencing, mRNAseq libraries were made in triplicate using total RNA from WT and Nxt1 larvae and the ScriptSeq RNA-Seq Library Preparation Kit (Illumina), followed by 100 bp paired-end sequencing on an Illumina HiSeq 2500. For larval carcass, mRNAseq stationary larvae were dissected for three replicates each of WT and Nxt1 larvae. Libraries were generated using the TruSeq Stranded mRNA Library Prep (Illumina), and sequenced with 2x75bp paired-end with the NextSeq 500/550 Mid Output v2 kit, yielding a range between 6.4 and 11.1 M reads per sample for whole larva and 21–32 M reads per sample for larval carcass. For larval carcass, Ribo-zero total RNAseq, the TruSeq Stranded total RNA-seq with Ribozero gold LT kit (Illumina) was used. The circRNAseq preparation was as for total RNAseq, but with the addition of an RNAse R treatment step before library synthesis. These libraries were pooled in equimolar proportions and sequenced on a 2x75bp high-output NextSeq500 cartridge, yielding 43–50 M PE reads per sample of total RNA and 31–36 M reads per RNAseR treated sample (Suppl data file: CircRNA_data.xlxs).

Whole larval and carcass mRNAseq data were analyzed using Tuxedo suite, including Cuffdiff to identify differentially expressed genes, as well as alternative splicing and isoform expression (Trapnell *et al*. 2012). Total RNAseq was aligned to the genome with STAR v.2.5.3a, read counts were generated using Subread package FeatureCounts v. 1.6.2. (Dobin *et al*. 2013; Liao *et al*. 2014) DESeq2 (Love *et al*. 2014) was used to normalize reads counts and calculate fpkm values. CircRNAs were identified using PTESFinder v.1 (Izuogu *et al.* 2016). For differential expression analysis we required a structure to be detected in at least two biological replicates, and be supported by at least 10 back-spliced junction spanning reads across the three biological replicates. We then calculated BPM and averaged this across the three replicates before comparing the genotypes. The mock (non RNAseR treated) replicates were used to quantify the spliced RNA (but not necessarily poly adenylated) and pre-mRNA of the host transcript. As a measure of circRNA abundance, we used the number of back-splice per million mapped reads (BPM), as defined by (Haque *et al.* 2020) (see Supplementary Methods). This measure is conceptually similar to fpkm values from RNA-seq and comparable between samples.

### Data availability

RNA-seq datasets for *w1118* and *Nxt1* transheterozygotes larvae are available via GEO: whole larvae before, during, and after puparium formation (GSE125781), and late third instar larval carcass samples (mRNA: GSE125776; total RNA and CircRNA: GSE135591). The identified circRNA structures, read counts, and calculated BPMs are given in Supplementary file CircRNA_data.xlsx. 

Supplementary material is available figshare DOI: https://doi.org/10.25387/g3.13366643.

## Results

### *Nxt1* mutant pupae are frequently curved, have uneverted spiracles and fail head eversion

**Figure 1:**
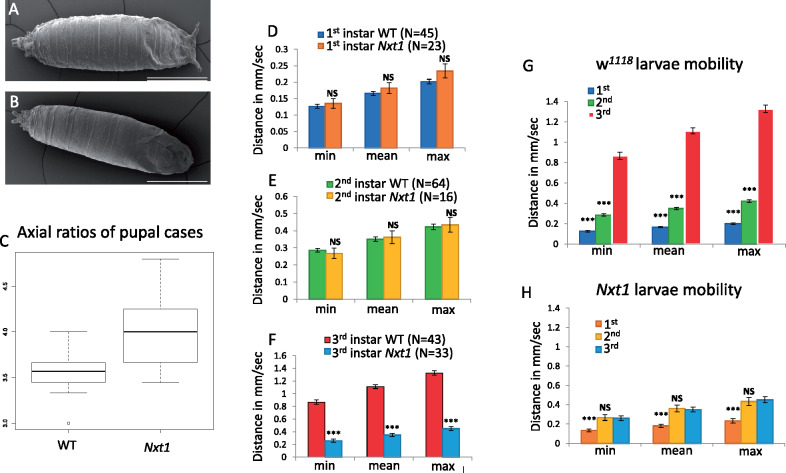
Morphological defects in Nxt1 trans-heterozygote pupae and reduced mobility of 3rd instar larvae. (A, B) Scanning electron microscopy shows normal spiracles in wild type (A) and uneverted spiracles in *Nxt1* (B) mutant pupal cases. (C) Axial ratios show *Nxt1* mutants are thinner and longer than wild type. (D–F) Mobility analysis of 1st, 2nd and 3rd instars of wild type and *Nxt1* trans-heterozygotes. (G–H) All 1st, 2nd, and 3rd instar data combined for each genotype. Student’s *t*-test ***p value ≤ 0.001. ns: not significant.

We have previously described that *Nxt1^z2-0488^/Nxt1^DG05102^* transheterozygotes are male and female sterile, but also have significantly reduced viability (Caporilli *et al.* 2013). *Nxt1^DG05102^* is a null allele caused by a P-element insertion into the coding sequence of the gene, while *Nxt1^z2-0488^* is a hypomorphic allele that disrupts a hydrogen-bonding network in the core of the protein thus reducing protein stability (Caporilli *et al.* 2013). Many *Nxt1^z2-0488^/Nxt1^DG05102^* transheterozygote pupae had a curved shape and uneverted spiracles. To quantify this morphological defect, we measured the axial ratios from the pupa and confirmed that the mutant pupae were significantly longer and thinner than wild type (Figure 1C) (*t*-test, *P* = 1e−08). Scanning electron microscopy (SEM) revealed that the surface structure of the pupal case was similar between mutant and wild type (Figure 1, A and B) while in Nxt1 trans-heterozygotes, 50% larvae (*N* = 36) had uneverted anterior spiracles. We have also previously reported that the *Nxt1^z2-0488^/Nxt1^DG05102^* transheterozygote pupae show failure in head eversion (Caporilli *et al.* 2013). Air bubble formation and migration are implicated in head eversion and therefore the viability of the pupa. We filmed *w^1118^* and *Nxt1^z2-0488^/Nxt1 ^DG05102^* trans-heterozygote pre-pupae and found that in mutant pupae the air bubble formed as normal, however the migration to the anterior of the pupa often failed; a typical series is shown (Supplementary Figure S1). Only about 20% (*N* = 182) of the Nxt1 trans-heterozygote pupae survived through to adulthood, compared to 90% (*N* = 287) viability for control *w^1118^* pupae (Supplementary Figure S2). We assessed the progression of pupal development at four time points (24, 48, 72, and 96 h pupa), found a dramatic drop in viability after 48 h (Supplementary Figure S2).

### Muscle degeneration occurs in *Nxt1* mutant larvae

Muscle contractions, triggered by an ecdysone pulse, are required at the initial stage of pupariation, as the larvae contract longitudinally, evert their spiracles and become white pre-pupae (reviewed in Yamanaka *et al.* 2013). A further muscular contraction of the prepupa partially withdraws the anterior tracheal lining (Shatoury 1959) and an air bubble forms in the abdominal cavity. The air bubble is forced to the anterior by abdominal muscular contractions, creating space inside the puparium to evert the head, which up to this point has been developing internally (Shatoury 1959).

To understand whether a transcriptional defect downstream of ecdysone signaling is responsible for the phenotype, analogous Nxt1's role in regulation of testis-specific transcripts via the transcriptional activation complex tMAC (Caporilli *et al.* 2013), we performed RNA sequencing of pooled whole larvae before, during, and after the pulse of ecdysone. Of 87 ecdysone-responsive genes (Fletcher and Thummel 1995), only four were mildly misregulated (2-4x down: *Eig71Eb, Eig71Ef*, and *Eig71Eg;* 2x up: *Eip63F-1*). Mutants of these four genes do not show an air bubble phenotype (Wright *et al.* 1996; Vaskova *et al.* 2000), therefore, it is unlikely that failure of expression of ecdysone-responsive genes is responsible for the Nxt1 air bubble phenotype.

To determine whether the defects in *Nxt1* mutants are due to muscular defects, we characterized the structure and function of muscles in larvae. A larval movement assay was used to track 1st, 2nd, and 3rd instar larvae to compare their speed between stages and between *w^1118^* and Nxt1 trans-heterozygotes. There was no significant difference in the average speed of mutant larvae compared to wild type at either 1st or 2nd instar stage (Figure 1, D and E). Normally, wild-type 3rd instar larvae travel up to 5× faster than 2nd instars (Figure 1G). In contrast, Nxt1 trans-heterozygous 3rd instar larvae were significantly slower than control animals (Figure 1F), indeed, they were no faster than 2nd instar larvae (Figure 1H), despite being much larger.

**Figure 2:**
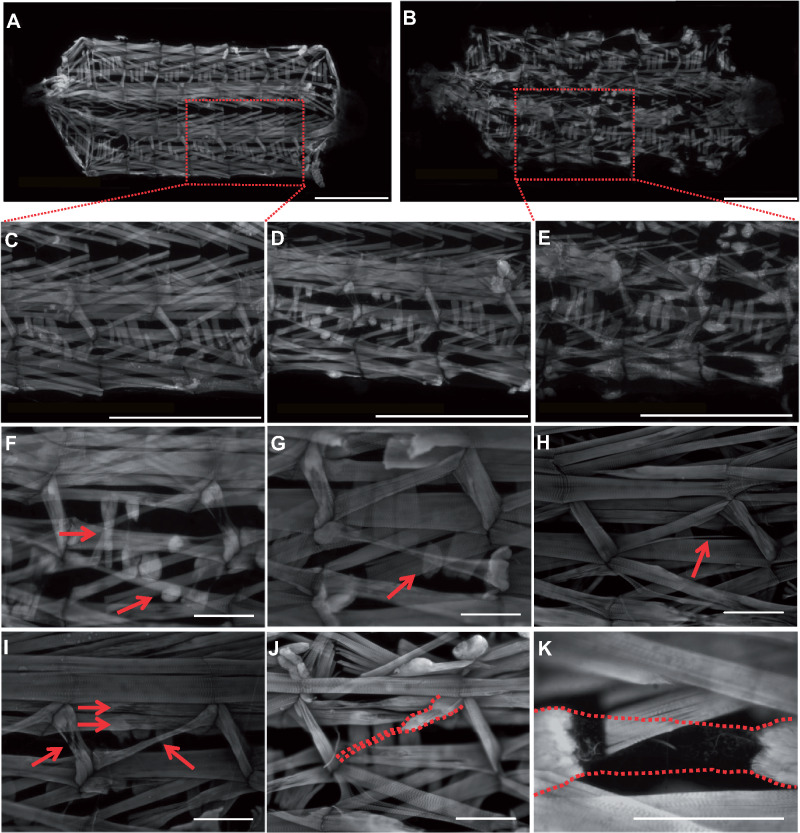
*Nxt1* trans-heterozygote 3rd instar larvae show muscle degeneration. Muscles of larval carcass preparations (WT A and C, *Nxt1* mutants B and D–K) imaged with FITC-phalloidin. (A) Overview of wild type 3rd instar larva muscles. (B) Overview of *Nxt1^DG05102^*/*Nxt1^Z2-0488^* 3rd instar larva muscles. (C) Higher power image of larva hemisegments from (A). (D) Hemisegments from *Nxt1^DG05102^*/*Nxt1^Z2-0488^* with mild muscle degeneration. (E) Higher power image of larva hemisegments from (B). (F) Lateral transverse (LT) 1 and 2 crossover (short arrow). (F–G) Short LT4 muscle (long arrow). (H) Lateral Oblique (LO) 1 fiber split (red arrow). (I, J) LO 1 partially degenerated into a small bundle of fibers connected to the segment border muscle (SBM). (K) Dorsal oblique (DO) 2 showing strings of fibers (red dotted lines) attached to each end of the muscle. Samples orientated from posterior (left) to anterior (right). (A–E) Scale bar = 1 mm. (F–K) Scale bar = 200 μm.

To investigate the muscle structure in the mutant animals, we stained of 3rd instar larval body wall muscles with phalloidin which labels F-actin. The stereotypical pattern in A2–A7 of 30 muscles per hemisegment (Bate 1990) was clearly observed in wild type (Figure 2, A and C), however, *Nxt1^z2-0488^/Nxt1^DG05102^* trans-heterozygotes frequently showed muscle degeneration (Figure 2, B, D, and E). Quantitation of 6 animals, and 240 muscles per animal, revealed variability between individuals and included thinner muscles (15%), loss of sarcomeric structure (22%), degenerating muscles (77%), torn muscles (8%) (Figure 2, F–K); all control animals had no defects.

**Figure 3:**
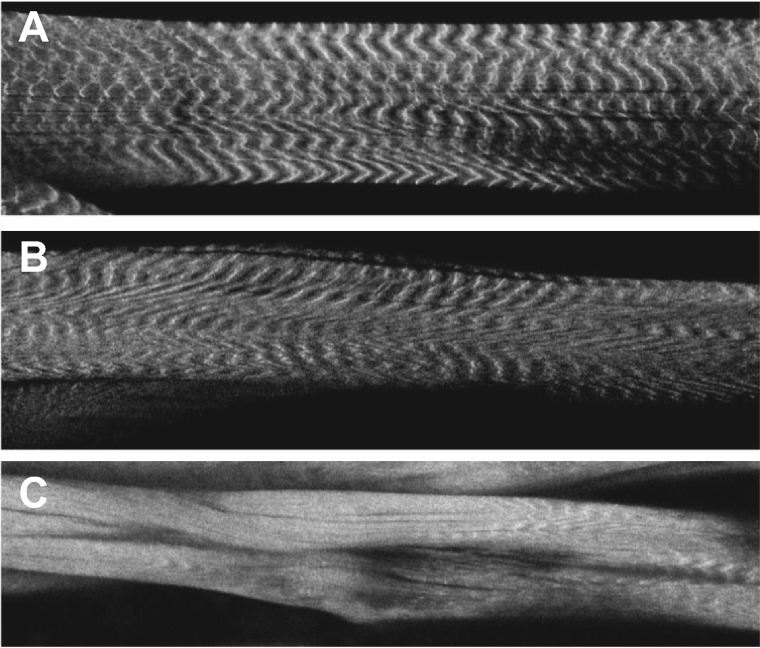
Sarcomere structure compromised in degenerating muscles. (A) Wild-type muscles stained for actin with phalloidin show normal thin filaments. (B) *Nxt1* trans-heterozygotes non-degenerating muscles with normal thin filaments. (C) *Nxt1* trans-heterozygotes degenerating muscles with compromised thin filaments; the normal ribbed structure is dramatically altered.

*Nxt1^z2-0488^/Nxt1^DG05102^* trans-heterozygote mutant animals also sometimes had defects in internal muscle structure. Normal muscle sarcomere structure consists of thick and thin filaments, and phalloidin staining of actin reveals this structure by labeling the thin filaments in the sarcomere (Figure 3A). For Nxt1 trans-heterozygotes, the sarcomere structure was compromised in 22% of the muscles examined. These muscles showed more uniform phalloidin staining indicating weak or absent differentiation of thin *vs* thick filaments (Figure 3C). This muscle degeneration and sarcomere structure defect phenotype were not fully penetrant, and some Nxt1 trans-heterozygotes had more normal sarcomere structure and less obvious muscle degeneration. This is consistent with the finding that about 20% of the mutant third instar larvae are able to develop to adulthood (Figure 3B). First and second instar larvae had normal musculature, and thus the defects are due to degeneration rather than a developmental defect.

**Figure 4:**
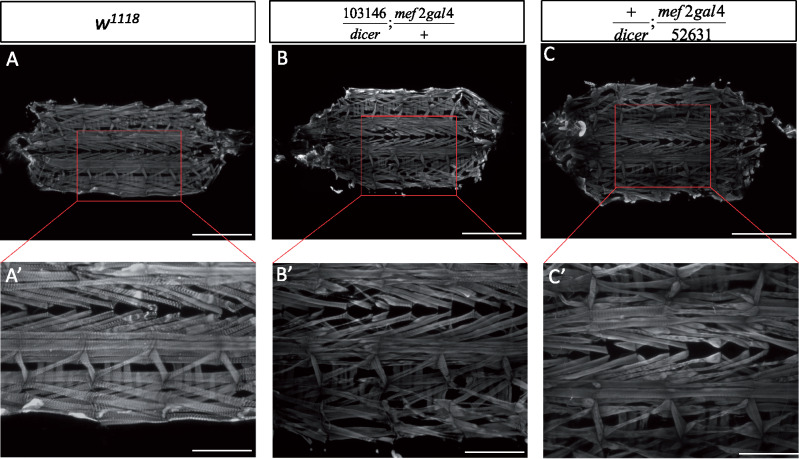
Early 2nd instar muscle degeneration with *Nxt1* RNAi. (A–A’) Wild-type 2nd instar larvae carcass preparations, stained with phalloidin, showing normal muscle composition and no damage. (B–Bʹ) Signs of muscle degeneration with the RNAi 103146 line driven by Mef2-Gal4 visible in early 2nd instars with thinner muscles and fiber damage (see insert Bʹ). (C–Cʹ) Similar degeneration defects are shown for the RNAi 52631 line. (A–C) Scale bar = 1 mm. (Aʹ–Cʹ). Scale bar = 0.5 mm.

To confirm that the muscle phenotype observed was due to defects in *Nxt1* rather than being a nonspecific effect, we used Mef2-Gal4 to drive UAS-RNAi expression specifically in muscles. We combined this with UAS-dicer and high induction temperature to achieve a strong knock down of the transcript. Both RNAi lines 103146 (chromosome 2) and 52631 (chromosome 3) had previously been shown to effectively knock down *Nxt1* and phenocopy the mutant phenotype in spermatocytes (Caporilli *et al.* 2013). Phalloidin staining of these 2nd instar larvae showed extensive muscle degeneration (Figure 4). When the temperature was 25°C for embryonic development, with a shift to 29°C after hatching, line 52,631 still induced early 2nd instar lethality, while 103,146 gave a weaker phenotype of third instar larval lethality, very similar to the *Nxt1^z2-0488^/Nxt1^DG05102^* trans-heterozygote hypomorphic condition. This indicates that knock down of *Nxt1* specifically in muscles is able to phenocopy the *Nxt1* mutant situation, and shows that the degeneration is due to reduction in *Nxt1* activity in muscles.

**Figure 5:**
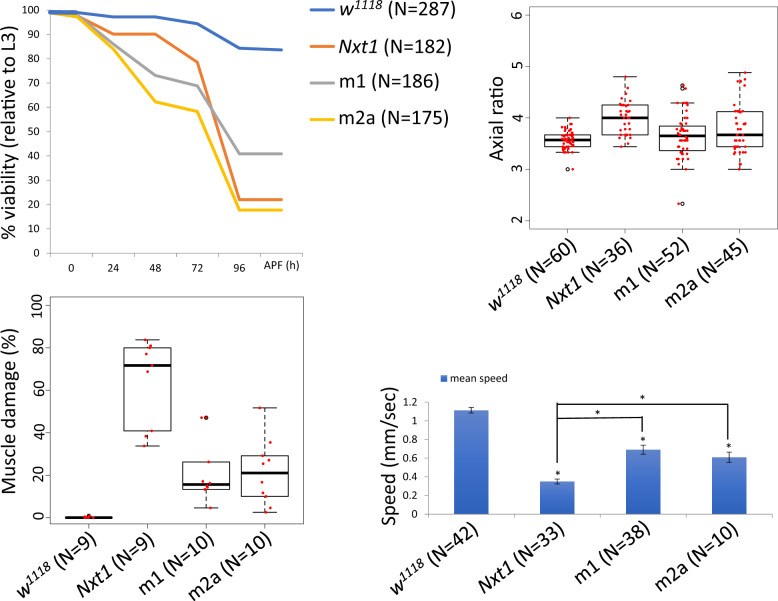
Rescue of *Nxt1* trans-heterozygote phenotypes. *Nxt1* transheterozygote defects in viability (A), pupal morphology (axial ratios of pupae, B), muscle morphology (C) and 3rd instar motility (D) were partially rescued by over-expression of GFP-Nxt1 in muscles (Mef2-Gal4); m1 and m2a are two independent UAS-Nxt1 insertions, full genotype of these animals is *w; Nxt1^DG05102^ UAS-Nxt1/Nxt1^z2-0488^; Mef2-Gal4/+.*

We expressed GFP-Nxt1 via the UAS-gal4 system both exclusively in muscles at a high level (with mef2-gal4>UAS-GFP-*Nxt1*) and ubiquitously at a lower level (with arm-gal4>UAS-GFP-*Nxt1*) in *Nxt1* trans-heterozygotes. High level, muscle-specific, and GFP-Nxt1 expression was able to partially rescue muscle integrity in *Nxt1* trans-heterozygotes (∼13–26% with two 47% outliers (*N* = 10) Figure 5C) and partially rescue the pupa lethality (∼40% (*N* = 186); Figure 5A). On average, the axial ratios were similar to wild type, albeit more variable (Figure 5B). Finally, mobility was increased compared to *Nxt1* trans-heterozygotes, but was still significantly reduced compared to wild type (Student’s *t*-test *p < 0.05; Figure 5D). Lower level ubiquitous expression of GFP-*Nxt1* in Nxt1 trans-heterozygotes resulted in improved, but not fully rescued muscle integrity (∼3–33%) damage with one 73% outlier (*N* = 9), increased the pupa viability [∼75% (*N* = 163)] and increased larval mobility. The axial ratios were similar to wild type (Figure 5B).

### Muscle degeneration is associated with larval growth

**Figure 6:**
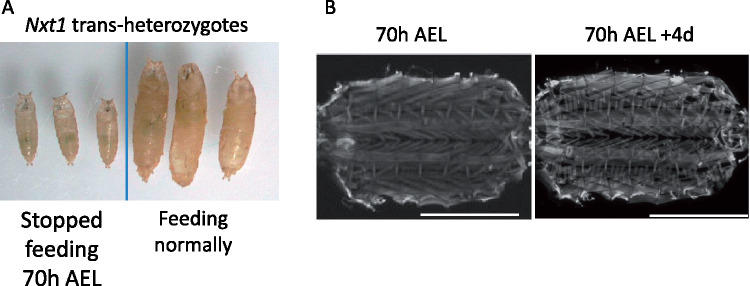
*Nxt1* trans-heterozygote muscles do not degenerate when larval growth is prevented. (A) *Nxt1* trans-heterozygote pupae were smaller, but had normal morphology when food was withheld from 70 h AEL. Three food-deprived and three normal feeding animals are shown. (B) *Nxt1* trans-heterozygote 70 h AEL were examined for muscle degeneration. Individuals that had not pupated four days after no feeding still had intact muscles (*N* = 10).

During the last instar phase, larvae grow substantially compared to earlier instars. Larvae removed from the food 70 h after egg laying do not grow, but still crawl until the normal time for pupation (∼112 h AEL) and may survive through to adulthood, generating very small flies (Beadle *et al.* 1938). Larvae at 70 h AEL are late 2nd, or early 3rd instars. To test whether growth or use (movement) is implicated in the muscle degeneration in *Nxt1* mutants, we fed wild-type larvae then starved them. 14-25% of the larvae removed from food at 70–73 h were able to pupate although less than 5% of those pupae were able to emerge as adults. We simultaneously examined Nxt1 trans-heterozygote larvae that were starved from 70-h AEL and found that few larvae pupated and none emerged as adults (similar to *w^1118^*). The pupae that formed had everted spiracles (Figure 6A) and resembled the wild-type controls. Interestingly, the larvae that failed to pupate were able to survive for several additional days as larvae before dying. Nxt1 trans-heterozygote larvae were dissected 4 days after they were removed from the food, and were stained with phalloidin. For all larvae (*n* = 10), no muscle degeneration was observed (Figure 6B). These larvae had been moving normally and thus using their body wall muscles for the four days of starvation. The lack of abnormalities in these animals indicates that the muscle growth, or high levels of force generation associated with third instar larval movement, rather than use *per se*, is critical for degeneration in *Nxt1* mutants.

### Other RNA export factors are also required for the maintenance of muscle integrity in *Drosophila* larvae

**Figure 7:**
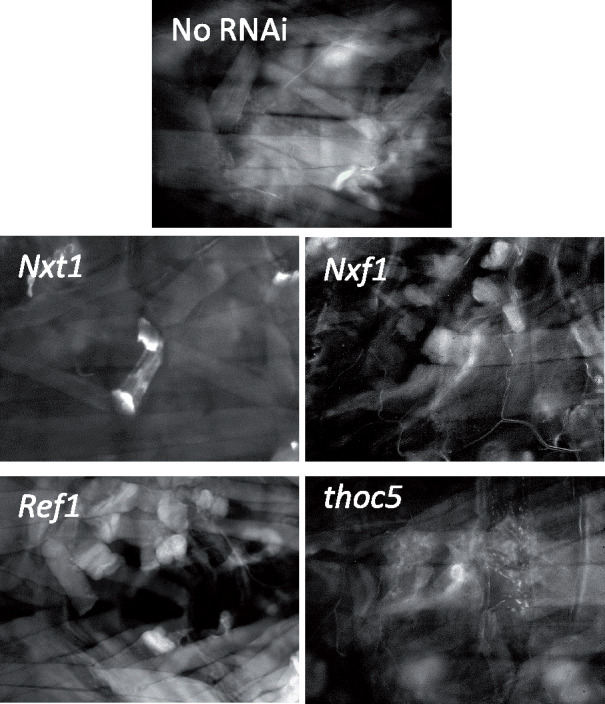
Muscle degeneration caused by RNAi against other RNA export pathway genes. (A) Control arm-gal4 driver line late 3rd instar larvae muscles stained with phalloidin shows normal muscle structure. (B–E) Muscle degeneration was observed after ubiquitous, moderate, induction of RNAi against *Nxt1* (B), *Nxf1* (C), *Ref 1* (D), and *thoc5* (E).

To determine whether the muscle degeneration phenotype is caused by defects in the RNA export pathway, we used arm-gal4 to drive RNAi hairpin constructs targeted against other RNA export pathway genes, specifically *thoc5, sbr (Nxf1), Ref1* and *Hel25E (UAP56)*. This is designed to reduce, but not completely eliminate the RNAi target gene expression throughout the larva; these. As a positive control, we used RNAi against *Nxt1*, and as a negative control we used both *w^1118^* and arm-gal4 alone. RNAi against *Hel25E* caused early larval lethality, even when tested at lower temperature, so we were not able to assess the role of this gene in muscle maintenance. For all the other genotypes, we assayed third instar muscle integrity with phalloidin staining, and found a similar phenotype to knock down of *Nxt1*, with muscle thinning and tearing apparent (Figure 7, B–E). Control larvae (arm-gal4 alone and *w^1118^*) had normal musculature (Figure 7A and data not shown). This indicates that the role of *Nxt1* in muscle maintenance is most likely to be attributable to its role in the RNA export pathway, although not necessarily for RNA export *per se*, rather than an unrelated function.

### Genes with long introns that also produce circRNAs are sensitive to the loss of Nxt1

**Figure 8:**
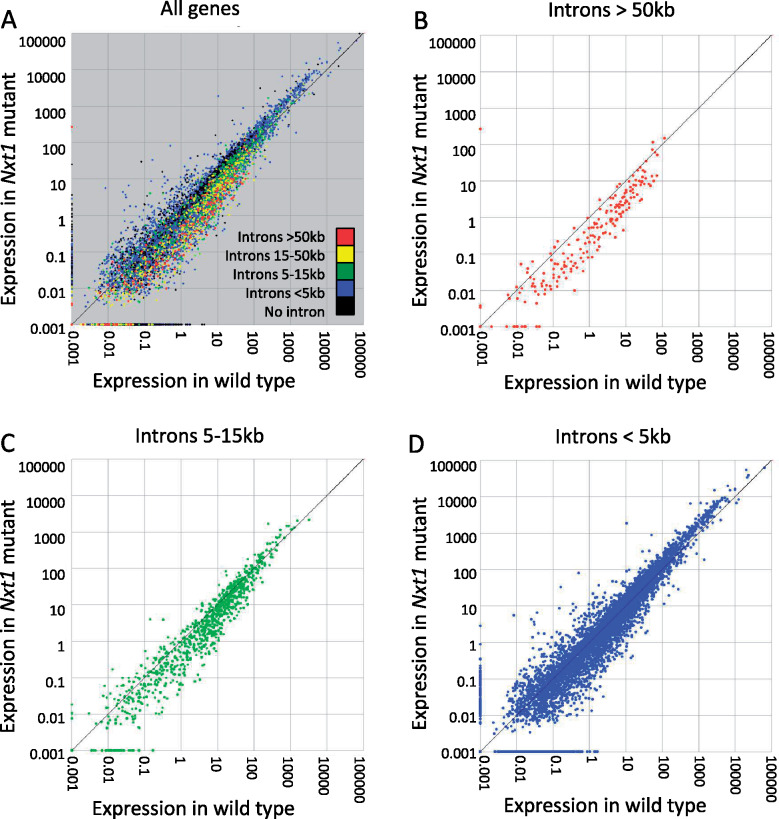
Genes with long introns are under-expressed in *Nxt1* trans-heterozygotes. mRNA level (FPKM) showing relative expression in WT and *Nxt1* larval carcass samples. (A) All genes are colored to show total intron length. Genes with long introns are typically expressed at lower levels in the mutant than in the wild-type sample. (B) Genes (red in A) whose total intron length is >50 kb, (C) Genes (green in A) whose total intron length is between 5 and 15 kb, (D) Genes (blue in A) whose total intron length is <5 kb.

The known role of *Nxt1* in RNA export and transcriptional control suggests that the muscle phenotype could be due to transcriptional or post-transcriptional defects in gene expression. To identify somatically expressed differentially expressed genes, we performed mRNA sequencing from stationary third instar larval carcasses (comprising primarily muscle and cuticle). Cuffdiff was used to reveal potential significant differences in overall transcript level and in alternative splicing. 572 genes were 2× or more upregulated in mutant compared to wild type, while 1340 genes were similarly down-regulated (Table 1). Only minor differences in alternative splicing were found, so we concentrated on the dramatic differences in mRNA expression levels, and looked for properties that could explain the overall gene expression changes. Transcripts from genes with long introns are reduced in EJC mutants (Ashton-Beaucage *et al.* 2010). In contrast, in *Nxt1* trans-heterozygote testes, transcripts from short and intron-less genes were dramatically reduced (Caporilli *et al.* 2013). We initially analyzed the total intron length, number of introns, and smallest/largest intron. This revealed that, in direct contrast to the situation in testes, genes down-regulated in *Nxt1* mutant carcass had more introns and a higher total intron length than nondifferentially expressed genes. Upregulated genes had fewer introns and a lower total intron length than the genes that were not differentially expressed [Mann–Whitney test *p*-value <0.05, except for 16-fold upregulated (*p* = 0.16)]; (Table 1; Figure 8). We extended the analysis by also looking at the gene length and shortest/longest transcripts (Supplementary Table S1). Again, a clear trend showed that down-regulated genes had longer median mRNA length and upregulated genes had shorter median mRNA length when compared to nondifferentially expressed genes (Mann–Whitney *p* < 0.05). Finally, the genes down-regulated in *Nxt1* mutants have more annotated mRNA isoforms than those upregulated or not differentially expressed (Supplementary Table S1). qRT-PCR for 12 genes with long introns confirmed the down-regulation seen in the RNA sequencing data (Supplementary Figure S3).

**Table 1:** Total intron lengths for genes down and upregulated from larval carcass sequencing

Median	No. of genes	Total intron length	Number of introns	Shortest intron	Longest intron
>1.5× down	1,821	5,769	9	59	2,341
>2× down	1,340	7,594	9	59	2,931
>4× down	567	10,201	11	59	3,642
>16× down	32	15,254	10	63	8,081
>1.5× up	1,339	368	3	62	160
>2× up	572	352	3	62	155
>4× up	89	237	2	62	133
>16× up	15	238	2	61	180
Unchanged	4,754	497	4	60	228

Genes with long introns are sources of circular RNAs, with many circular RNA exons being flanked by long introns (Jeck *et al.* 2013). We therefore compared the genes down-regulated in *Nxt1* muscles with lists of genes known to produce circRNA transcripts (Westholm *et al.* 2014). This well-curated list was generated from over 100 highly sequenced libraries from several *Drosophila* tissues. We found a striking overlap: 85% (466 of the 567) genes 4x or more down-regulated in *Nxt1* muscles had at least one read consistent with a circRNA. 199 of these genes were in the higher confidence set associated with at least 10 circRNA reads. 57 of the 186 genes that were at least 8× down-regulated in *Nxt1* muscles were also in the high confidence circRNA set. As a comparison, only 25% of genes over fourfold upregulated in Nxt1, 30% of nondifferentially expressed genes and 36% of all *Drosophila* genes have been shown to produce circRNA transcripts (Westholm *et al.* 2014). When using our muscle circRNA dataset, we detected circRNAs from 34% of the genes 4× or more down-regulated at the mRNA level despite detecting circRNAs derived from only 6% of all Drosophila genes. We did not detect any circRNAs from either wild type or mutant samples derived from genes that are 4× or more upregulated in the mutants.

CircRNAs are predominantly produced from genes that are known to have neuronal functions and whose expression is higher in nervous system, even when the analysis is performed on tissues outside the nervous system (Westholm *et al.* 2014). Consistent with this, embryonic expression pattern enrichment analysis via FlyMine revealed that the genes down-regulated in *Nxt1* muscles are indeed significantly enriched for expression in nervous system [such as ventral nerve cord (*P* = 1.13e−8), embryonic brain (*P* = 3.06e−6)]. We conclude that Nxt1 is important not only for the export of mRNAs but also for normal expression of mRNAs from genes with long introns, particularly those that also produce circRNA products.

### CircRNAs are reduced in *Nxt1* mutants

mRNAs and circRNAs are mutually exclusive products made from the same primary transcript. If Nxt1 and the RNA export pathway controls the ratio between mRNAs and circRNAs, down-regulation of *Nxt1* would lead to an increase in circRNA products. Alternatively, Nxt1 could be implicated in the stability of the primary RNA to ensure normal levels of both products, in which case we would expect a reduction in circRNA in target genes in the mutants. In a third model, Nxt1 could act after completion of splicing to stabilize just the mRNA; in this case, the circRNA levels would not change in the mutant compared to wild type. To determine which of these models is most likely, we sequenced both total RNA (after depletion of the ribosomal RNAs) and circRNA (the RNAse R-resistant fraction) from larval carcass samples from wild type and *Nxt1* transheterozygotes. Library preparation did not include selection for polyadenylated transcripts, and thus allowed us to examine pre-mRNA, both spliced and unspliced (via reads that map to introns), as well as circRNA. Mutant and wild-type libraries were prepared in triplicate and sequenced to similar depths (∼34 M reads per RNAseR sample, ∼46 M reads per mock sample).

We identified circRNA structures via the junction-spanning back-spliced reads. We found a reduction in total circRNA abundance in *Nxt1* mutants; the ratio of reads in the total RNA sequencing data from back-spliced junctions compared to total mapped reads was significantly lower in the mutant sample than in the control sample (Chi-squared = 366, 1 df, *P* < 0.00001). To determine whether this effect was a very large change in abundance of a few circRNAs, or was due to changes in many distinct circRNAs, we counted the total number of circRNA structures identified in all our libraries. Taking together all replicates of both genotypes, we identified 3414 circRNA structures (with at least one read in one replicate) corresponding to 1101 genes. We detected more unique circRNA structures in all the WT samples (both total and RNAseR treated) than in the comparable mutant samples (Table 2). CircRNAs are rare, and datasets are typically noisy; to concentrate only on the high confidence structures, we imposed a detection threshold such that we only considered structures detected in at least two biological replicates, and supported by at least 10 junction-spanning reads in RNAseR treated samples. We found 523 different, backspliced, structures in wild type compared to only 404 in *Nxt1* samples, despite very similar library read depth. This suggests a reduction in the diversity of circRNA structures made in the mutant muscles. About 303 structures, derived from 256 genes, passed the expression threshold in both samples.

**Table 2:** Circular RNA structures identified in RNAse R and mock-treated samples

		*Nxt1*			WT		
		rep1	rep2	rep3	rep1	rep2	rep3
# CircRNA structures identified	RNAseR treated	710	773	802	986	817	997
	Mock	82	140	141	236	199	195
Total # CircRNA structures identified^a^	RNAseR treated	1,576			1,864		
	Mock	290			460		
# High confidence CircRNA^b^	RNAseR treated	404			523		
	Mock	10			25		

a # CircRNA structures identified is every structure detected, including those in only one replicate.

b # High confidence CircRNA is number of structures supported by at least 10 back-spliced junction reads and detected in at least two replicates.

**Figure 9:**
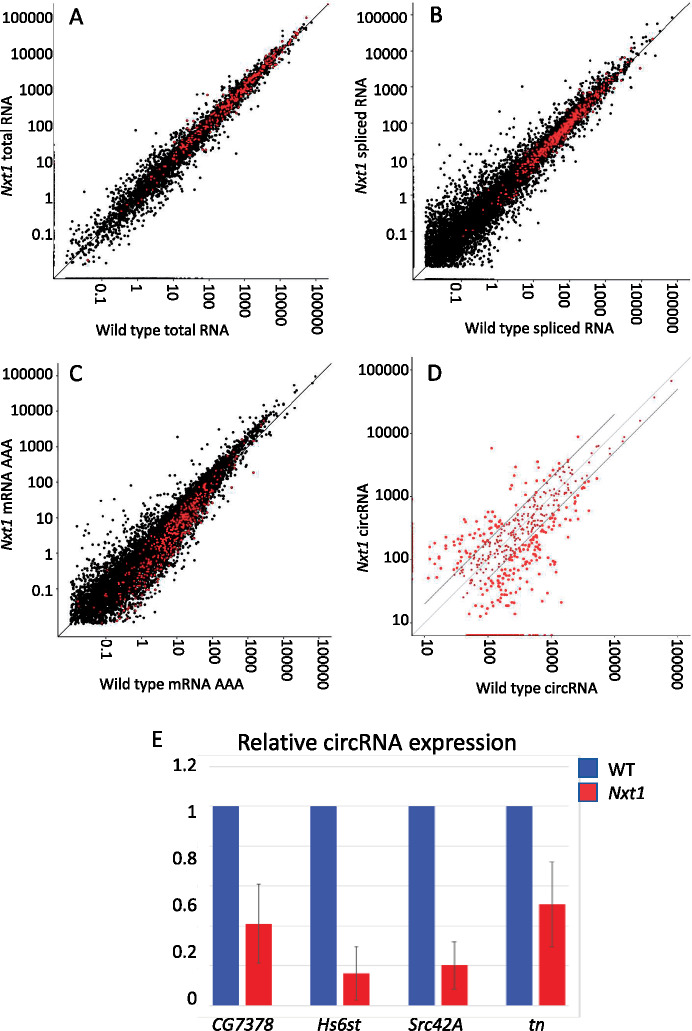
Reduced expression of circRNAs but not nascent transcripts in *Nxt1* trans-heterozygotes. Expression of nascent RNAs (A) and spliced mRNA that is not necessarily polyadenylated (B) is similar in total RNA samples from WT and mutant larval carcasses. Genes that produce the circRNAs shown in (D) are highlighted in red. (C) Sequencing only polyadenylated transcripts reveals a reduction in the expression of the mature mRNA from the genes that produce circRNAs. (D) circRNAs detected by sequencing after RNAseR digestion are reduced in mutant larval carcass compared to WT. BPMs for all structures that passed the threshold in either genotype are plotted. Structures twofold differentially expressed between mutant and wild type are highlighted. (E) Q-RT-PCR reveals reduced expression of the major circRNA products from *CG7378* (exons 2–5), *Hs6st* (exons 3–5), *Src42A* (exon 2), and *tn* (*abba*) (exon 7) in *Nxt1* trans-heterozygotes compared to wild-type controls. Expression levels were calculated from three biological replicates, normalized using rp49 mRNA as a reference control. Error bars show 95% confidence intervals.

We compared the expression levels of the more abundant circRNAs, *i.e.* those that passed the expression threshold in one or both genotypes, comprising 624 structures. This revealed that the expression of these circRNAs is on average significantly reduced in *Nxt1* compared to wild type (Wilcoxon signed rank test, *P* = 0.0005). Significantly more individual structures were reduced in expression than increased in expression in the mutant; 162 structures were detected but were 2× or more down-regulated in the mutant compared to wild type; a further 106 structures that passed the expression threshold in wild type were not detected at all in the mutant sample. In contrast, 83 structures were 2× or more up-regulated in the mutant compared to wild type, and 49 structures that passed the expression threshold in mutant were not detected at all in wild type (Figure 9D).

For the genes that produced circRNAs above the expression threshold, pre-mRNA levels (assessed via intron reads in the total RNA sample) were typically unchanged between wild type and mutant (highlighted red in Figure 9A). We also found no difference between levels of spliced RNAs in the total RNA samples (highlighted red in Figure 9B), although we did detect reductions in the polyadenylated mRNA levels (via mRNAseq) in mutants (highlighted red in Figure 9C).

We validated the reduced expression of circRNA products from four genes, *CG7378*, *Hs6st*, *Src42A*, and *tn* by q-RT-PCR, using primers that flank the back-spliced exon–exon junction, and that therefore only amplify cDNA derived from the circRNA (Figure 9E). *t*-tests confirm that reductions in expression for all four of these samples were significant (*N* = 3, *P* ≤ 0.01 in all cases). Supplementary Figure S4 shows RNAseq data aligned to the genome annotation for the four validated circRNA-producing genes, showing the transcript region that contributes to the circRNA. We conclude that the role of Nxt1, in addition to ensuring that mRNAs are exported from the nucleus, is to ensure post-transcriptional stabilization and processing of transcripts from genes with long introns. For many genes, the effect of reduction in this activity is that there is reduced expression of the final processed RNAs from these genes—both mRNAs and circRNAs.

### Increased expression of *abba* rescues the muscle degeneration, but not semilethality

**Figure 10:**
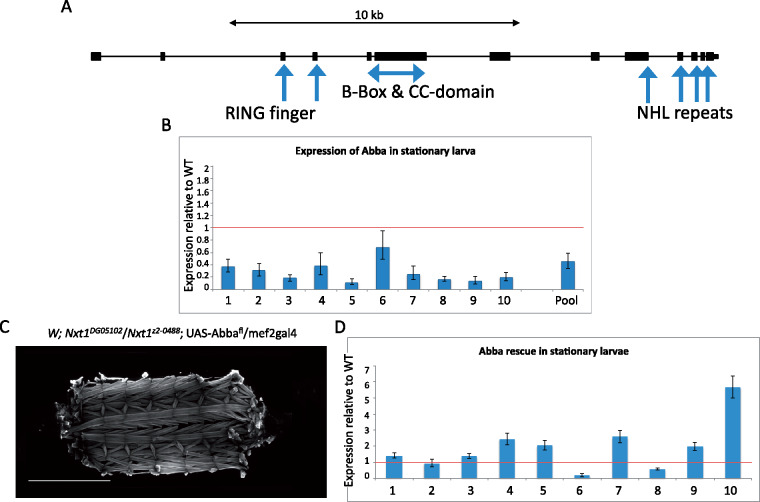
Increasing *abba* expression rescues Nxt1 trans-heterozygote muscle degeneration. (A) Genomic structure of the longest *abba* transcript including all known domains, exons are broad boxes, introns are narrow lines. (B) Q-RT-PCR analysis of expression of *abba* in 10 individual stationary larvae plus a mix of 30 stationary larvae in Nxt1 trans-heterozygotes, compared to a mix of 10 and 30 stationary wild-type larvae, respectively. (C) Increasing *abba* with the UAS/Gal4 system specifically in muscles rescues muscle degeneration, as revealed by FITC-phalloidin staining. (D) Expression of *abba* in 10 individual larvae from the rescue experiment, compared to a mix of 10 stationary wild-type larvae. Rp49 was used for normalization.

Having identified a global defect in mRNA and circRNA for many genes, we were interested in understanding how this links to the muscle degeneration phenotype. Inspecting the RNA expression of known muscle-specific or muscle-enriched genes in our original whole larva mRNA-seq data, revealed that *abba* [also known as *thin* (*tn*)] was significantly down-regulated. Abba is an essential TRIM/RBCC protein that maintains the integrity of sarcomeric cytoarchitecture (Domsch *et al.* 2013). *abba* loss of function mutations are lethal, with the larvae and pupae being long and thin and having muscle degeneration (LaBeau-DiMenna *et al.* 2012). *abba* is a large gene with several long introns, which also produces a circRNA derived from circularization of exon 7 (Supplementary Figure S4D). qRT-PCR confirmed the reduction in *abba* expression (*n* = 30 larvae per sample; Figure 10). Levels of *abba* mRNA were consistently highly variable between individual stationary larvae in both wild type and mutants (Figure 10) but significantly down in 9 out of 10 larvae, when compared with a pool of 10 WT larvae (Figure 10B).

To restore *abba* expression in the mutants, we used an UAS-*abba*^full length (fl)^ cDNA construct (kindly gifted by Hanh T. Nguyen; hereafter referred to as UAS-*abba*) and expressed it under the muscle-specific driver mef2-gal4 in the *Nxt1* trans-heterozygote background. Staining of the muscles of these larvae with phalloidin, showed that muscle degeneration was fully rescued in 12 out of 16 animals and partially rescued in the other four (Figure 10C). qRT-PCR of individual stationary larvae again showed high variability between individuals, but 8 out of 10 had *abba* expression equal to or exceeding that seen in the pool of wild type control larvae (Figure 10D).

The finding that the expression of just one gene, *abba*, is sufficient to restore normal muscle function is surprising given the large number of genes whose expression is altered in the mutant larvae. This genetic rescue experiment indicates that the muscle defect in *Nxt1* mutant larvae is primarily due to the reduction in *abba* mRNA expression. However, the pupal lethality of most *Nxt1* transheterozygotes is not solely caused by the muscular degeneration, since we found that the survival to adulthood was not rescued by increased *abba* expression (22%; *N* = 187). RNA processing defects leading to reduced expression of the many other target genes is likely associated with a reduction in the viability of the animal.

## Discussion

### Reduction in Nxt1 function causes muscle degeneration

Nxt1 is primarily known for its role in the RNA export pathway. Nxt1 binds to Nxf1, which interacts with the nuclear pore complex for exporting mRNA to the cytoplasm (Herold *et al.* 2001). Reducing Nxt1 protein level reduces the Nxt1-Nxf1 dimer and without Nxt1, Nxf1 interacts less effectively with the nuclear pore complex (Wiegand *et al.* 2002). Since most mRNAs are exported via the Nxt1–Nxf1 route, it is expected that many transcripts will be affected, and indeed this is what is seen in tissue culture cells (Herold *et al.* 2001). Consistent with this, homozygotes of the null *Nxt1* allele are embryonic lethal. The hypomorphic allele, we have used, causes reduced protein stability (Caporilli *et al.* 2013), but retains sufficient Nxt1 activity to support mRNA export, and thus allows us to explore other functions of this protein. Intriguingly, the transheterozygotes were able to develop apparently normally to the third instar larval stage, and to pupate. Most lethality occurred at this transition, with obvious defects in air bubble migration and pupal shape. The phenotype was not fully penetrant, and some Nxt1 trans-heterozygote pupae had normal morphology; 20% of transheterozygote third instar pupae became adults.

The defects in pupal morphology were caused by muscle degeneration during the third instar larval stage. Second instar larvae had normal muscle pattern, morphology, and mobility. This indicates that the embryonic establishment of the larval musculature is not affected by the reduction in *Nxt1* in the hypomorphic allele. In zebrafish, a specific isoform of NXT2 (an Nxt1 family member) was implicated in heart development in early embryogenesis. In this situation however, the heart patterning and initial structure were abnormal, rather than the heart forming normally and then degenerating (Huang *et al.* 2005).

Muscles comprise tandem arrays of sarcomeres containing thick and thin filaments, arranged in myofibrils. *Nxt1* transheterozygote larvae presented a variety of defects including muscle atrophy (thinning) and loss of integrity (tearing and splitting). Frequently, we saw a loss of filamentous actin in the middle of the muscle while the ends had f-actin; very occasionally we saw balls of muscles associated with loss of attachment or catastrophic failure of muscle integrity and muscle severing. Even when the muscle shape was unaffected, we found a loss of normal internal architecture with disruption of the sarcomeric arrays. It is likely that the reduction in larval mobility, the axial ratio defect, the failure of spiracle eversion, and the failure of air bubble movement are all a direct result of the degeneration of muscles in the mutant larvae. All these processes require efficient and coordinated muscle contractions, which are compromised by a lack of Nxt1. More degeneration, and thus less contraction, on one side of the animal would explain the curvature of many mutant pupae.

During the larval phase, the muscles increase in fiber size ∼50-fold. This dramatic growth occurs without the addition of new cells; increased DNA content is achieved via endoreduplication, and increased muscle volume is driven by cell growth associated with new myofibril and sarcomere assembly (Piccirillo *et al.* 2014). The majority of this growth occurs in third instar larvae, and depends on nutrient supply and sensing of this via the insulin/Akt/Tor pathway (Demontis and Perrimon 2009). Deficits in muscle growth have nonautonomous effects on the growth of the whole larva (Demontis and Perrimon 2009). We did not detect any difference in the overall size of third instar mutant larvae, indicating that the defects are not due to defects in nutrient supply or sensing. When *Nxt1* hypomorphic larvae were starved from the late 2nd or early 3rd instar larval stage they remained alive and continue to move, but growth was blocked. This treatment was sufficient to rescue the muscle degeneration. Thus, the primary cause of the degeneration is defective (re)-organization during muscle growth, rather than damage caused by use of the muscles, although we cannot rule out that the higher forces required for third instar larval movement are not also implicated.

RNAi constructs to disrupt Nxt1 function in muscles caused degeneration, without pattern defects, even in second instar larvae, indicating that the precise level of Nxt1 function is critical. Very high level, muscle specific, RNAi construct expression, driven from embryonic stages, presumably reduces the level of active Nxt1 in muscles in embryos and early larval stages even beyond that found in the hypomorphic allele combination. In this situation, there would be reduced Abba both during embryonic muscle formation and at the early growth stages, and this would impact the muscle integrity even before the extensive third instar larval growth period. Driving *Nxt1* RNAi ubiquitously but at a lower level, phenocopies *Nxt1* transheterozygotes, with muscle degeneration in third instars. Driving RNAi targeted against other RNA export pathway factors ubiquitously, with arm-gal4, also phenocopies Nxt1 transheterozygotes. Importantly, these experiments, coupled with the partial rescue of the muscle defects by expression of Nxt1, confirm that the phenotype is caused by reduction in Nxt1 function rather than by a different gene or by a neomorphic effect of the point mutation in *Nxt1^Z2-0488^*. Additionally, these experiments confirm that the muscle maintenance phenotype is not caused by a moonlighting function of the protein in a different biological process, but rather that muscle maintenance is particularly sensitive to the reduced function of the whole RNA export pathway.

### Muscle degeneration can be rescued by increasing *abba*

We were surprised that a hypomorphic allele of a pleiotropic factor such as Nxt1 had such a specific phenotype of muscle degeneration. We reasoned that, while Nxt1 is important for the normal expression of many genes, the muscle defects could result from the reduction in just one or a few crucial genes. A precedent for a pleiotropic factor being involved in many functions but essential for just one comes from analysis of PP1β. While many proteins can be dephosphorylated by this enzyme, its only essential target is Sqh, a myosin regulatory light chain. Reduction in the amount of active myosin, for example, by loss of one copy of the nonmuscle myosin heavy chain gene (*zip*), can rescue the lethality caused by loss of *PP1β* (Vereshchagina *et al.* 2004). Our initial RNA-seq of whole larvae was a mixed sex population, and unfortunately the majority of genes differentially expressed between samples were those with testes-specific or testis-enriched expression. This is consistent with our previous findings that *Nxt1* is critical for expression of genes regulated by tMAC in testes (Caporilli *et al.* 2013), but meant that the signal from relatively mild expression changes from somatic tissues was less apparent. Nevertheless, one gene, *abba*, with a known role in muscles stood out as being mildly down-regulated in stationary larvae. The phenotype of *abba* mutants is strikingly similar to that we describe for *Nxt1* hypomorphs, particularly with thinner muscles, long thin pupae, and defects in both spiracle eversion and air bubble migration (LaBeau-DiMenna *et al.* 2012; Domsch *et al.* 2013). Abba is a TRIM/RBCC protein involved in maintaining the integrity of sarcomeric cytoarchitecture (LaBeau-DiMenna *et al.* 2012; Domsch *et al.* 2013), and in the *Drosophila melanogaster* homologue of human *Trim32*, defects in which cause limb-girdle muscular dystrophy 2H (Frosk *et al.* 2002). Trim32 is localized to the costamere, which overlies the Z-disk and ensures attachment of the sarcomere to the overlying extracellular matrix via the dystrophin–glycoprotein complex.

The phenotypes of defective sarcomere structure, fraying, and muscular atrophy are all consistent with the reduction in *abba* (costamere) function. The growth of muscles involves the generation of new sarcomeric units, each requiring a new costamere and thus new Abba production and incorporation. The gradual decline in muscle integrity seen during growth may be attributable to reduction in Abba protein levels meaning that the stability of newly formed sarcomeres is reduced. Indeed, in mouse, the expression of Trim32 is upregulated in muscles that are remodeling (Kudryashova *et al.* 2005). Consistent with this model, increasing expression of *abba* using a cDNA construct driven with the Gal4/UAS system was sufficient to rescue the muscle defects seen in *Nxt1* mutants. Starvation presumably maintains muscle integrity by blocking muscle growth, so no new sarcomeric units need to be added. The rescue by a cDNA also indicates that it is the reduction in *abba* mRNA, rather than a reduction in *abba* circRNA that is important for the muscle phenotype, as the cDNA construct cannot generate the circRNA.

### *Nxt1* is particularly important for the expression of mRNAs and circRNAs from genes with long introns

Transcripts with many and large introns are more sensitive to the loss of *Nxt1*, akin to the loss of the EJC (Ashton-Beaucage and Therrien 2011). In direct contrast, genes without introns were particularly sensitive to loss of *Nxt1* in testes (Caporilli *et al.* 2013). This difference can be explained by tissue-specific differences in the efficiency of expression of genes with introns, and the roles of introns in alternative transcript processing decisions in the nucleus. In somatic cells, expression of genes without introns is much less efficient than the expression of genes with introns; relatively few genes with introns are expressed in typical somatic cells, and the median mRNA level of these genes is lower than the median mRNA level derived from genes with introns. The male germline, however, is particularly efficient at the expression of intron-less genes (Caporilli *et al.* 2013). The requirement for *Nxt1* for mRNA expression of specific genes in testes was linked to a feedback system, whereby *Nxt1* promoted transcription of genes regulated by testis meiotic arrest complex (TMAC) and also promoted the stability of transcripts produced from these genes (Caporilli *et al.* 2013). The majority of intronless genes expressed in the testis require TMAC for their transcription.

We found no evidence in our RNAseq of carcass samples for a feedback loop from the RNA export factors to the transcriptional machinery analogous to the system in testes. RNA-seq data from larval carcasses, which is highly enriched for larval muscles, therefore allowed us to examine the role of Nxt1 in post-transcriptional regulation of RNAs in a somatic tissue in which we have shown its function is critical, without this complication of a feedback to a specific transcription factor complex. This revealed a strong correlation between changes in mRNA expression in *Nxt1* mutants and intron length. For down-regulated genes, the more dramatic the down-regulation, the longer the total intron length. Similarly, for upregulated genes, the more upregulated the gene, the shorter the total intron length. Transcripts with many introns may require more Nxt1–Nxf1 dimer recruitment to ensure normal processing and stability. If TREX loading is less efficient on transcripts with long introns, particularly if these are being processed to make circRNAs in addition to mRNAs, then these transcripts could be preferentially sensitive when the availability of Nxt1 protein is limiting.

Circular RNAs are a relatively recently described class of RNAs that are produced as alternative products from the same primary transcripts as mRNAs. Global analysis of circRNA abundance reveals that many genes can encode circRNAs, but the propensity for a gene to produce a circular transcript is related to intron length, particularly those that flank the back-spliced exon(s) (Jeck *et al.* 2013). Our RNA-seq analysis suggests that the mature mRNAs from genes that also produce circRNAs is reduced in Nxt1 mutants, while the primary transcript is not reduced. We found that the diversity of circRNAs is lower in the mutants, and that, for those detected in both WT and mutant, the circRNAs levels are on average reduced in the mutants. Mutation of EJC components has also been shown to have a differential effect on mRNA production from genes with long introns (Ashton-Beaucage *et al.* 2010). At least some genes down-regulated in the EJC knock down also had aberrant splicing patterns (Ashton-Beaucage *et al.* 2010). In contrast, we found few defects in alternative splicing in *Nxt1* mutants. However, it is also interesting to note that 249/315 genes down-regulated after knock down of the EJC were also on the list of genes that produce at least one circRNA, and 99 are on the higher confidence list of 10 or more circRNAs (Ashton-Beaucage *et al.* 2010; Westholm *et al.* 2014). In tissue culture cells, reduction of Hel25E (UAP56), an essential factor in the RNA export pathway has been shown to reduce total expression of the assayed circRNA by about 20%, and to cause a nuclear accumulation of longer circRNAs (Huang *et al.* 2018). The RNA export pathway has also been shown to be linked to 3' end processing and poly adenylation. TREX subunit THOC5 is recruited to target transcript 3'UTRs by poly adenylation specific factor 100 (Tran *et al.* 2014). ALYREF association with RNAs is promoted by the 5' cap, by the EJC, and, crucially, also in the 3' UTR by nuclear polyA binding protein (PABPN1) (Shi *et al.* 2017). In light of this, it is likely that the EJC and the export adapter Nxt1 (and other factors in the RNA export pathway) are particularly important in processing of transcripts that are alternatively spliced to produce mRNA and circular RNA outputs. We found that pre-mRNA levels of these target genes are not reduced in the mutants, while mature, poly adenlyated mRNAs are, suggesting that export factors act late during processing and 3'end formation.

Human muscular dystrophies are inherited genetic conditions that cause muscles to weaken, leading to an increasing level of disability. Some, such as Duchenne muscular dystrophy and limb girdle muscular dystrophy 2H, are caused by mutations in genes encoding muscle structural proteins (*dystrophin* and *Trim32*, respectively) (Hoffman *et al.* 1987; Frosk *et al.* 2002), while others, like myotonic dystrophy, are caused by defects in RNA metabolism (reviewed in Wheeler and Thornton 2007; Meola and Cardani 2015). For example, DM1 myotonic dystrophy, a trinucleotide repeat in the 3' UTR of the *DMPK* gene results in sequestration of the splicing regulator MBNL1, and this in turn causes defects in muscle gene expression, particularly alteration of splicing patterns. Interestingly, both MBNL1 and *muscleblind* (*mbl*), the *Drosophila* ortholog of MBNL1, produce abundant circular RNAs (Ashwal-Fluss *et al.* 2014). *mbl* is expressed in both muscles and the nervous system, and is one of the many circRNA-producing genes whose mRNA is reduced (approximately 10×) in *Nxt1* mutant larvae. The production of the *mbl* circRNA is regulated by Mbl protein itself, in an autoregulatory feedback loop (Ashwal-Fluss *et al.* 2014). In vertebrates, it appears that MBNL1 does not regulate the production of its own circRNA in neuronal cells, but the effect in muscle has not been determined (Konieczny *et al.* 2017, 2018).

Our discovery that defects in an RNA export factor, *Nxt1*, can generate a muscular dystrophy phenotype and is associated with differential expression of specific mRNAs and circRNAs in *Drosophila melanogaster* suggests that investigation of the RNA export pathway, and circRNAs, in the context of the disease is warranted.
